# MDR-ER: Balancing Functions for Adjusting the Ratio in Risk Classes and Classification Errors for Imbalanced Cases and Controls Using Multifactor-Dimensionality Reduction

**DOI:** 10.1371/journal.pone.0079387

**Published:** 2013-11-13

**Authors:** Cheng-Hong Yang, Yu-Da Lin, Li-Yeh Chuang, Jin-Bor Chen, Hsueh-Wei Chang

**Affiliations:** 1 Department of Electronic Engineering, National Kaohsiung University of Applied Sciences, Kaohsiung, Taiwan; 2 Department of Chemical Engineering and Institute of Biotechnology and Chemical Engineering, I-Shou University, Kaohsiung, Taiwan; 3 Division of Nephrology, Department of Internal Medicine, Mitochondrial Research Unit, Kaohsiung Chang Gung Memorial Hospital, Chang Gung University College of Medicine, Kaohsiung, Taiwan; 4 Department of Biomedical Science and Environmental Biology, Kaohsiung Medical University, Taiwan; 5 Cancer Center, Kaohsiung Medical University Hospital, Kaohsiung Medical University, Kaohsiung, Taiwan; University of Granada - Q1818002F, Spain

## Abstract

**Background:**

Determining the complex relationship between diseases, polymorphisms in human genes and environmental factors is challenging. Multifactor dimensionality reduction (MDR) has proven capable of effectively detecting statistical patterns of epistasis. However, MDR has its weakness in accurately assigning multi-locus genotypes to either high-risk and low-risk groups, and does generally not provide accurate error rates when the case and control data sets are imbalanced. Consequently, results for classification error rates and odds ratios (*OR*) may provide surprising values in that the true positive (TP) value is often small.

**Methodology/Principal Findings:**

To address this problem, we introduce a classifier function based on the ratio between the percentage of cases in case data and the percentage of controls in control data to improve MDR (MDR-ER) for multi-locus genotypes to be classified correctly into high-risk and low-risk groups. In this study, a real data set with different ratios of cases to controls (1∶4) was obtained from the mitochondrial D-loop of chronic dialysis patients in order to test MDR-ER. The TP and TN values were collected from all tests to analyze to what degree MDR-ER performed better than MDR.

**Conclusions/Significance:**

Results showed that MDR-ER can be successfully used to detect the complex associations in imbalanced data sets.

## Introduction

Genome-wide association studies (GWAS) can detect the several single nucleotide polymorphisms (SNPs) associated with genotype frequencies of cases and controls that have significant effects on disease susceptibility [1], [2], [3], [4], [5], [6]. Although GWAS provides representative SNPs from the entire genome, many SNPs with a low or marginal significance are frequently excluded. However, most SNPs discovered by GWAS still have effects on disease susceptibility when association effects amongst SNPs are considered; they may play a role in determining disease susceptibility due to gene-gene interaction. SNP association effects are believed to have important implications for the human disease risk [7], [8], [9]. The data mining and machine learning methods employed to gather the GWAS data pose significant computational challenges when simultaneously trying to evaluate the complex interactions amongst all tested SNPs. Different computational approaches have been developed to examine epistasis in family-based and case-control association studies [10], [11], [12], [13], [14], [15], [16], [17],[18],[19],[20],[21].

MDR was proposed by Ritchie et al. [22]. It is a non-parametric statistical method for the detection of high-order gene–gene and gene–environment interactions in case-control studies [22], [23]. The idea behind MDR is to classify the multi-locus genotypes into high-risk and low-risk groups to effectively reduce the genotype predictors from *n* dimensions to one dimension. MDR was shown to detect gene-gene interactions reasonably well for several disease phenotypes, including hypertension [24], [25], [26], bladder cancer [27], coronary artery disease [28], and autism [29]. Once an MDR attribute is constructed, it can be statistically evaluated using any classification method, e.g., naive Bayes, decision trees, or logistic regression [26]. Computational methods such as bootstrapping, cross-validation, and permutation testing can be employed as wrappers in MDR-based constructive induction and classification to facilitate identification of a best set of predictors and their statistical analysis model. Other improvements of the performance and applicability of MDR have been proposed, e.g., OR-MDR [30], GMDR [31], MBMDR [32], and other methods [33]. However, the majority of studies typically considers a balanced number of cases and controls. When a substantial imbalance is present in the data, problems arise in MDR. MDR has its weakness in accurately assigning multi-locus genotypes to either high-risk and low-risk groups, and does generally not provide accurate error rates when the case and control data sets are imbalanced. Consequently, results for classification error rates and odds ratios (*OR*) may provide surprising values in that the true positive (TP) value is often small.

In this study, we propose a classifier function based on the ratio between the percentage of cases in case data and the percentage of controls in control data into the MDR classifier in a method we called MDR-ER. This method assigns multi-locus genotypes and estimates the classification error to overcome the MDR shortcoming in imbalanced data sets. The MDR-ER method uses the ratio between the percentage of cases in the case data and the percentage of controls in the control data to weigh the disease risk, so that correct classification into high-risk and low-risk groups can be performed and the dimensionality significantly reduced. In addition, we use the percentage of cases in case data and the percentage of controls in control data to evaluate the classification error rate so that MDR can accurately select the smallest error rate amongst the SNP combinations. Hence, a reasonable true positive (TP) and true negative (TN) value can be obtained from a 2-way contingency table by using the large imbalance of cases to controls for each combination of genotypes.

The MDR method is briefly introduced in Section 2.1, and the MDR-ER method elaborated on in Section 2.2. An example giving a comparison between the MDR and MDR-ER results is provided in Section 3 using a data set obtained from the mitochondrial D-loop of chronic dialysis patients. A discussion and final conclusions are offered in Section 4.

## Methods

### MDR Method

**Figure 1:**
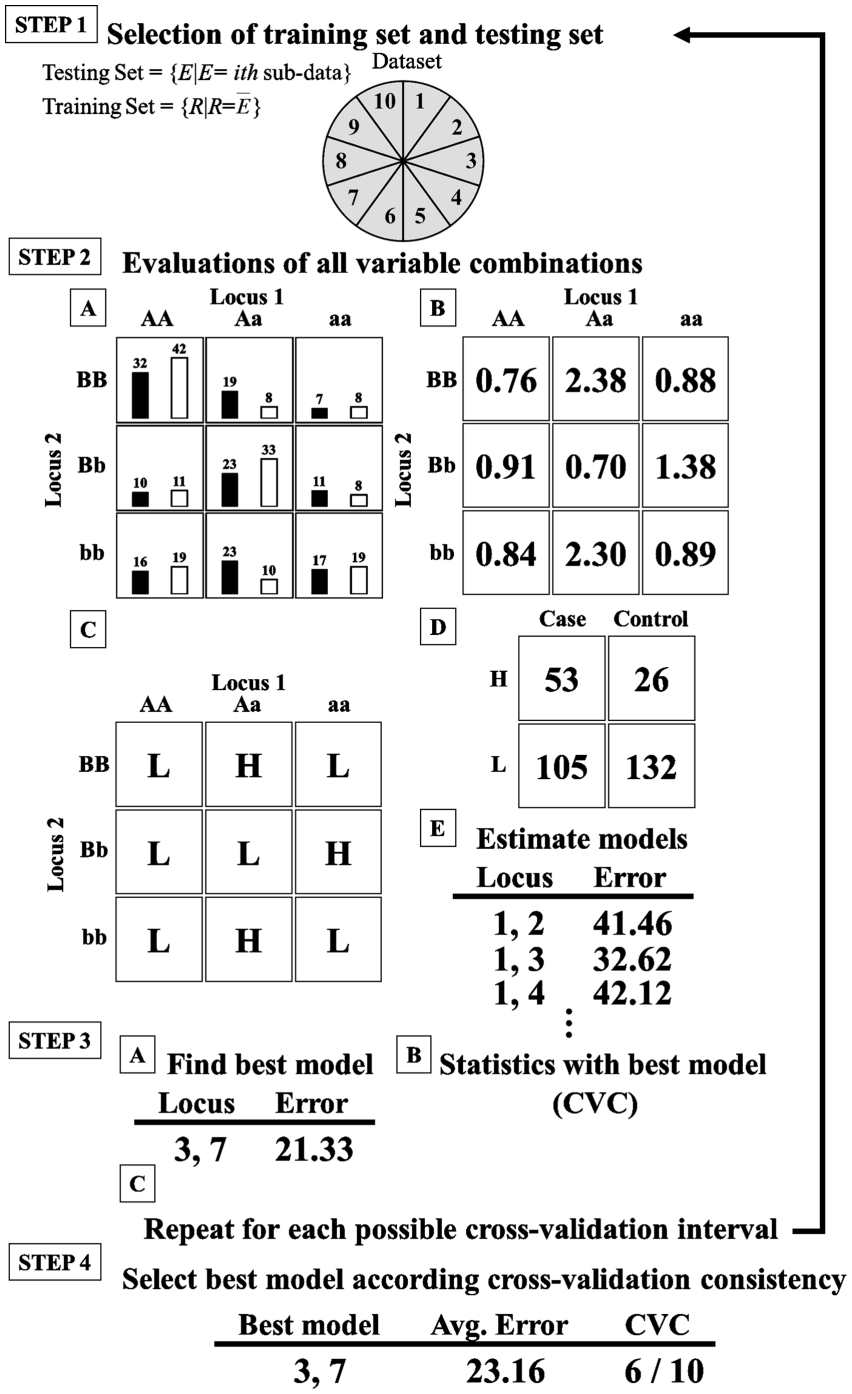
MDR flowchart.

The objective of MDR is to change the representation of the data space in such a way that detection of interactions can easily be performed. This is accomplished by combining two or more attributes into a single attribute that can be modeled using a discrete data classifier. Table 1 illustrates the MDR pseudo-code, and Figure 1 and File S1 illustrate the details of the MDR procedure. In a first step, the data are divided into 10 subsets for cross-validation (CV)–nine subsets are classified as training sets and one subset as an independent testing set. The training and testing sets are respectively defined as the vectors *R* and *E*, the elements of which represent samples in the data sets. In a second step, the value of *n* is designated depending on the number of factors being considered. Then, a set of *n* genetic and/or environmental factors is selected. The *n* factors (i.e., loci) and their possible multifactor classes are represented in the *n*-dimensional vector space.
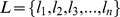
(1)


**Table 1:** MDR pseudo-code.

01: divide data into 10 subsets
02: **for** *D* = 1 **to** 10 subsets
03: classify *D* subset as the test data set and the other subsets as the training data set
04: **training data:**
05: **for** *N* = 1 **to** n-order combination of SNPs
06: **for** *C* = 1 **to** all combination of genotypes
07: determine the high/low risk groups in *C* cell
08: **end** *C*
09: compute the misclassification error
10: **end** *N*
11: choose the best combination with the minimum misclassification error
12: **end** training data
13: **test data:**
14: compute the prediction error of the best combination in the test data
15: **end** test data
16: collect the best combination into *B*≡(*B*_1_, *B*_2_, *…*, *B*_10_)
17: **end** *D*
18: compute cross-validation consistency from *B*
19: choose the best combination with the minimum prediction error

Next, the ratio of the number of cases to the number of controls within each multifactor class is calculated (step 2A). The black bar in Figure 1(2A) and Figure S1 in File S1 represents the cases and the white bar represents the controls. The ratio between cases and controls is evaluated by Eq. 2 (step 2B and Figure S2 in File S1).
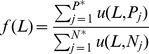
(2)where



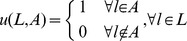
where the cases are labeled *P* and the controls are labeled *N*. *P* ^*^ and *N* ^*^ represent the sizes of case and control groups in the training set, respectively. *L* is a vector of variable combinations (as in Eq. 1). *u*() represents a match (given a score of “1”) if all parameters *l* in vector *L* match their cases or controls; a mismatch is given the score “0”.

Each multifactor class in the *n*-dimensional space is labeled ‘H’ if the ratio of the number of cases to that of the controls is equal to or exceeds a particular threshold; otherwise it is labeled ‘L’ (step 2C and Figure S3 in File S1). Hence, we can compute the four frequencies in a 2-way contingency table (TP, FP, TN, and FN) (step 2D and Figure S4 in File S1). The *n*-dimensional space is thus reduced to one dimension with two levels (high-risk and low-risk groups). Usually, the threshold is determined as the ratio of the number of cases to the number of controls in the training data set. The threshold is equal to the one in a balanced data set. Finally, in step 2E, the classification error is evaluated by Eq. 3.
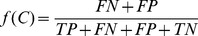
(3)


Among all the multifactor combinations, the MDR model with the lowest number of misclassified individuals is selected. In step 3, the model with the best misclassification error rate is selected and the prediction error rate of the model is estimated using the independent test data (step 3A). In addition, the best model in each cross-validation is collected and named the cross-validation consistency (CVC). With 10-fold cross-validation, the data are divided into 10 equal parts and the model developed on 9/10 of the data (i.e., the training data). After training, the remaining 1/10 of the data (i.e., the independent test data) is evaluated. This is repeated for each possible 9/10 and 1/10 of the data, and the resulting ten prediction error rates are averaged. Steps 1–3 are repeated for each possible cross-validation interval. Finally, the highest CVC with the lowest misclassification error is regarded as the best model (step 4). If a tie between 2 or more models occurs, then the model found first is regarded as the best model.

### MDR-ER Method

MDR uses the ratio between cases and controls to detect the high-risk and low-risk groups. Although the threshold *T* = 1 can be used to distinguish high-risk genotype combinations from low-risk genotype combinations, fault can be found with the imbalance of a data set. Unlike MDR, MDR-ER uses the ratio between the percentage of cases in case data and the percentage of controls in control data to detect the high-risk and low-risk groups. The percentages of cases and controls in data sets allow us to clearly detect the highest ratio in the case group and in the control group. We propose Eq. 4 to evaluate the ratio of case percentage to control percentage.
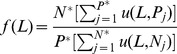
(4)where



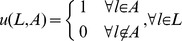



In Eq. 4 we define the cases as *P* and the controls as *N*. *P* ^*^ and *N* ^*^ represent the size of case group and the size of control group in the training set. *L* is a vector of variable combinations (as in Eq. 1). *u*() represents a match (given a score of “1”) if all parameters *l* in vector *L* match their cases or controls; a mismatch is given the score “0”. For example, the first cell shows 114 cases and 342 controls. Evaluation with Eq. 4 yields a value of 1.22, which means that the cell is assigned a low-risk label (704*114)/(193*342); *P* ^*^ and *N* ^*^ are 193 and 704, respectively (Table S1 in File S1).

The second function modifies the classification error rate of MDR. Eq. 5 was introduced into MDR by Velez [34]. The function computes the classification error rate based on the arithmetic mean of the sensitivity and specificity. Eq. 5 is introduced so that the two classes equally account for both positive and negative errors caused by the class imbalance. This balanced classification error is algebraically identical to the error rate when data sets are completely balanced.
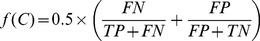
(5)


*C* represents a vector in a cell that includes the parameters TP, FP, FN, and TN. TP and FN represent the number of cases that are assigned to the high-risk and low-risk groups, respectively. FP and TN represent the number of controls that are assigned to the high-risk and low-risk groups, respectively.

The difference between the two methods lies in the fact that MDR-ER adjusts the amount of cases and controls from a number to a ratio. This provides a more robust model to accurately assign multi-locus genotypes to either high-risk or low-risk groups and provides an accurate classification error when the class variable has a different, i.e., imbalanced, ratio of cases to controls.

## Results

### Data Set

The data set was obtained from our previous chronic dialysis association study with mitochondrial SNPs in the D-loop region [35]. Information pertaining to the 77 SNPs in the data set are described in the literature [35]. In order to test the methods, the data sets were generated by random classification into *n* groups, where *n* is the number of cross-validations in MDR. Ten-fold cross-validation with 9/10 as a training set and 1/10 as a test set was used.

We generated a total of 100 different data sets. Over all 100 data sets, the ratio of controls (n = 704) to cases (n = 193) was 3.65∶1. In the 1000 training sets with 10-fold cross-validation, the range of ratios of cases to controls was 3.41 to 3.95; the mean (SD) ratio was 3.65 (0.10). In this study, each data set was used once to test all the methods.

### Detection

For 100 implementations, we summed up the frequencies of results, which were selected based on the cross-validation consistency (CVC) and the classification error rate in each test. The highest frequency is shown in the best candidate model and consistency column in Table 2. The true positive (TP), true negative (TN), accuracy, and odds ratio (*OR*) were evaluated by the best candidate model. We compare two MDR methods: (1) MDR-E which incorporates Eq. 5 in the MDR method, and (2) MDR-ER which uses both Eqs. 4 and 5.

**Table 2:** Estimated effect (odds ratio and 95% CI) from individual SNPs of 23 steroid hormone metabolisms and signalling-related genes on the occurrence of breast cancer in patients.

Methods	Best candidate model	Consistency	TP	TN	Accuracy	OR (95% CI)
2-locus						
MDR-E	55, 64	100/100	19	689	0.54	5.02 (2.50–10.07)
MDR-ER	40, 56	66/100	131	307	0.56	1.63 (1.17–2.29)
3-locus						
MDR-E	3, 55, 64	96/100	19	691	0.54	5.80 (2.81–11.98)
MDR-ER	21, 59, 64	26/100	111	393	0.57	1.71 (1.24–2.36)
4-locus						
MDR-E	5, 17, 43, 64	33/100	22	689	0.55	5.91 (3.00–11.63)
MDR-ER	21, 59, 64, 71	64/100	108	423	0.58	1.91 (1.39–2.64)
5-locus						
MDR-E	5, 17, 34, 43, 64	100/100	28	686	0.56	6.47 (3.49–11.97)
MDR-ER	8, 21, 31, 59, 64	22/100	80	538	0.59	2.29 (1.64–3.21)

This study detected associations from 2-locus to 5-locus genotypes; all detection results are shown in Table 2. All results are statistically significant (*P*<0.001). The MDR-E and MDR-ER methods show different results in the 2-locus to 5-locus groups. The TP and TN frequencies show that TN is clearly bigger than TP for all implementations of the MDR-E and MDR-ER methods. However, the TP of the MDR-E in the 2-locus to 5-locus groups are smaller than the respective TPs in MDR-ER, whereas the TNs of MDR-ER in the 2-locus to 5-locus groups are a lot smaller than in MDR-E. Note that the big difference between the total TP and the total TN is due to the imbalance of the data sets, and the decreased TN number is due to the fact that FP grows with the TP. Consequently, the high value for TN in MDR-E leads to a high odds ratio value, but the 95% CI region is very large, i.e., the accuracy of the odds ratio is poor. On the other hand, MDR-ER has a lower odds ratio (1.63 to 2.29), but the accuracy of the odds ratio is superior to the one of the other methods. Overall, the accuracy of MDR-ER in the 2-way contingency table is the highest of the four methods.

### Power

**Figure 2:**
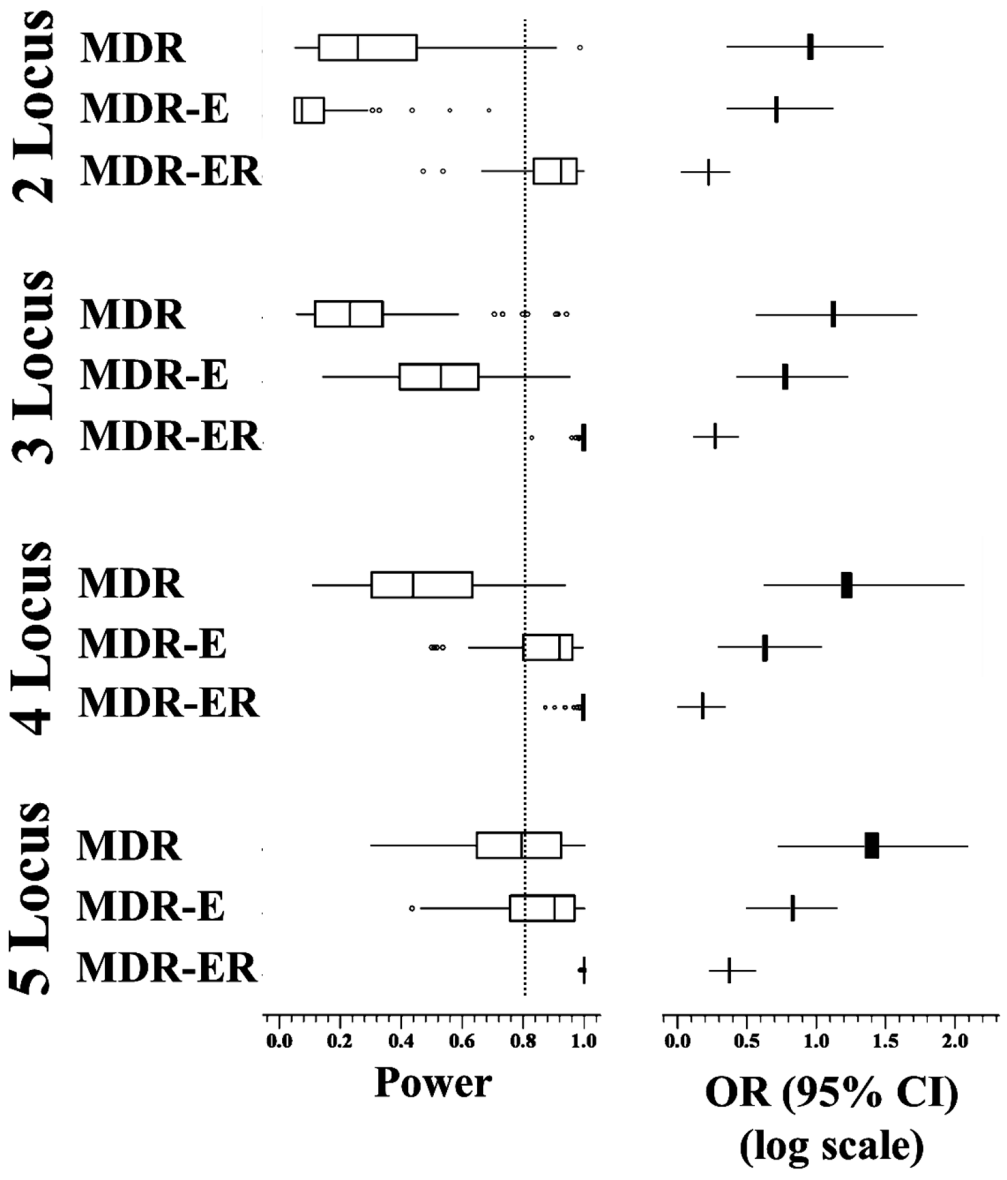
Power analysis of the four methods in 10-fold cross-validation.

The results of the test set can be used to examine the training set as to whether the model has a statistically significant difference for cases and controls or not. The results of the test set are usually not deemed statistically significant (at *P* = 0.001) during each cross-validation. Thus, we separate the results into two groups. The H0 group indicates that the result of the test set is the same as for the training set (H0), and the H1 group implies that the result of the test set is different from the training set. We use a power analysis to represent the degree of rejection for H0, i.e., 'power' is detection that is statistically significant (at α = 0.05). The statistical analysis was implemented by the G*power 3.1.5 tool. All power results are shown in Figure 2. It displays the extremes, the upper and lower quartiles, and the median of the power for MDR-E and MDR-ER on the 2- to 5-locus genotypes over 100 runs. The boundary of the box closest to zero indicates the 25th percentile, a line within the box marks the median, and the boundary of the box farthest from zero indicates the 75th percentile. Error bars left and right of the boxes indicate the 90th and 10th percentiles, respectively.

All boxes clearly indicate that the power is improved for high-locus genotypes. The power of MDR-ER is significantly higher than that of the other methods, and the stability of the power is good. The MDR-ER in 2- to 5-locus genotypes show a superior degree of power compared to MDR and MDR-E. The powers of MDR, MDR-E are usually smaller than 0.8. This means that the results have a high probability of not being statistically significant. Figure 2 shows the odds ratio and the 95% CI in the 2- to 5-locus genotypes. The boundary of the box closest to zero indicates the 25th percentile and the boundary of the box farthest from zero indicates the 75th percentile. Error bars left and right of the boxes indicate the minimum and maximum 95% CI over 100 runs, respectively. Comparison of the odds ratio shows that MDR-ER has the worst odds ratio of the four methods, but the smallest region between the upper and lower bounds of 95% CI.

## Discussion

MDR is a robust non-parametric method that detects nonlinear interactions amongst multiple discrete genetic factors. The advantage of MDR is that the representation of the data space can be changed so that high-order interactions can be computed by the statistical classifiers. However, MDR may not provide a robust enough model for the detection of nonlinear interactions when the ratio of cases to controls is imbalanced. Resampling techniques are usually used to improve MDR to detect epistasis in imbalanced data sets. However, we used an under-sampling technique to remove samples to arrive at the three different ratios (case:control 1∶1, 1∶2, and 1∶3), and then compared the real data set (1∶4) with these data sets. The results are shown in Tables S2 to S4 of the File S1. The best model in the 2- to 5-locus genotypes of both MDR-E and MDR-ER were not the same SNP combinations amongst the four data sets (1∶1, 1∶2, 1∶3, and 1∶4). This means that important information may be lost due to some samples being removed. Two different approaches can be used to improve MDR for classification when the data sets are imbalanced. The first is to find the optimal threshold, *T*, for determining the high-risk and low-risk in each cell, while the second approach defines a function for the classifier. However, finding the optimal threshold needed to train specific data sets is difficult. Defining a function for the classifier is more appropriate when trying to improve MDR for imbalanced data sets.

The objective of this study was to propose a function for improving the classifier of MDR when the number of cases and controls are not equal. The results show that our function successfully improved the MDR classifier in imbalanced data sets. We analyzed the details of the computation process to explain why MDR-ER is suited for imbalanced data sets. Table 3 shows detailed results obtained by running the whole data set for the 2-locus genotypes with MDR-E and MDR-ER. The first model shows the results (model, 55, 64) of MDR-E and the second model shows the results of MDR-ER (model, 40, 56). In the cell frequency column, the first number indicates the number of cases and the second number is the number of controls in each cell. In File S1 we show the computational process for the model SNPs (40, 56) from the results of MDR-E and MDR-ER at 2-locus combinations. Figure S2 in File S1 shows the sample distribution in the SNPs (40, 56); the number of control groups in all four cells is bigger than the number of case groups. The function, i.e., cases/controls, always computes ratio values smaller than the threshold *T* = 1 (Figure S2.A in File S1). On the other hand, Figure S2.B in File S1 shows the results of the proposed function computing the four cells; these results show that the numbers of high-risk (>1) and low-risk (<1) are balanced. This means that the function can improve the MDR classifier in imbalanced data sets.

**Table 3:** Analysis results of the difference between MDR-E and MDR-ER in 2-locus genotypes.

SNPs	Cell frequency^a^	MDR-E					MDR-ER				
		Strategy	Class	TP	TN	Error rate	Strategy	Class	TP	TN	Error rate
40, 56						0.5					0.44
	114∶342	0.33	Low-risk	0	342		1.20	High-risk	114	0	
	57∶280	0.20	Low-risk	0	280		0.74	Low-risk	0	280	
	5∶27	0.19	Low-risk	0	27		0.75	Low-risk	0	27	
	17∶55	0.31	Low-risk	0	55		1.13	High-risk	17	0	
Total				0	704				131	307	
SNPs											
55, 64						0.46					0.46
	174∶689	0.25	Low-risk	0	689		0.92	Low-risk	0	530	
	5∶4	1.25	High-risk	5	0		4.56	High-risk	5	163	
	14∶11	1.27	High-risk	14	0		4.64	High-risk	14	0	
	0∶0	0					0				
Total				19	689				19	689	

a the left number represents the number of cases and the right number represents the number of controls.

**Figure 3:**
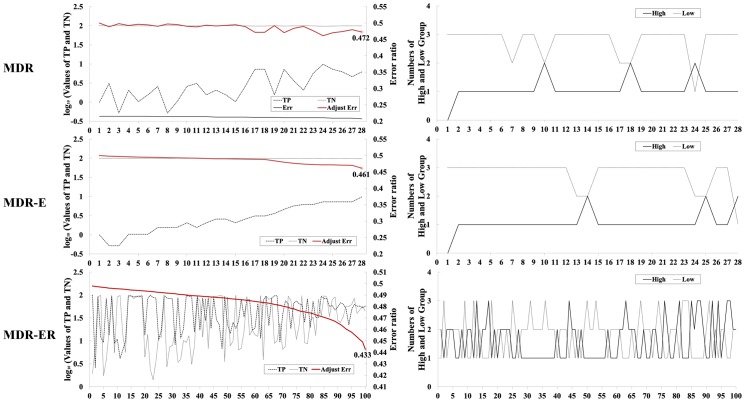
Frequency analysis of TP, TN, classification error, and numbers of high-risk and low-risk groups in 2-locus genotypes.

Figure 3 illustrates the advantage of Eqs. 4 and 5 for comparing the MDR, MDR-E and MDR-ER search processes. Figure 3 shows the 2-locus combinations with the TP, TN, error rate, and the number of high-risk and low- risk groups. The left figure illustrates the process of selecting the best model, and the right side illustrates the number of high-risk and low-risk groups in each model, respectively labeled “High” and “Low”. The horizontal axis represents the 2-locus combinations in the 100 different models, which are selected by systematic sampling from all models and sorted by error rate. The left and right figures show the same 100 models. The vertical axis represents the values of the above properties, in which the left scale in the left figure represents the log_10_ value for the proportions of TP and TN. For example, let the frequency of the high-risk group in cases (i.e., TP) be 131 and the case number 193. The proportion 67.88% is computed as 131/193×100%, and the log_10_ value is 1.83. The red line represents the error rate based on Eq. 5, denoted “Adjust Err”. In Figure 3, error rates of MDR in 28 models show values around 0.2; the number of TNs is much larger than the number of TPs. The number of TPs has increased in 28 models, but their error rates are not clearly improved. The sensitivity of the best model in MDR is 0.062, and the specificity is 0.993. On the other hand, MDR-E in Figure 3 clearly shows that TPs are improved by Eq. 5. However, the number of TNs is still larger than the number of TPs in all models. The right figure of MDR-E illustrates that the low-risk groups are bigger than the high-risk groups in 28 models. The sensitivity of the best model in MDR-E is 0.098, and the specificity is 0.978. For MDR-ER in Figure 3, the number of TNs is not always higher than the number of TPs, and thus it has good error rates which occur when the difference in the value for TP and TN is small. The right figure of MDR-ER illustrates that the difference between the number of high-risk groups and the number of low-risk groups is improved when we compare MDR and MDR-E. The sensitivity of the best model in MDR-ER is 0.679, and the specificity is 0.436. The distribution difference between high-risk and low-risk groups for an imbalanced data set is shown in Figure 3. In MDR and MDR-E, the cells are usually classified into low-risk groups due to the case number in each cell often being smaller than the control number. MDR-ER shows a good ratio of the number of high-risk groups and the number of low-risk groups. MDR-ER uses the Eq. 4 to calculate the ratio between cases and controls in all cells and effectively overcomes the poor identification of MDR for imbalanced data sets.

MDR-ER combines two functions to measure the low-risk and high-risk groups and evaluate the classification error to select the best model. Similar to the original MDR, MDR-ER is a non-parametric method and assumes no particular genetic model. However, the MDR-ER method has several advantages over the MDR method when imbalanced data sets are used. These are: (1) MDR-ER effectively classifies cells into high-risk and low-risk groups to increase the number of TPs, (2) the best model has a low error rate and a high sensitivity for disease prediction, (3) it only modifies the classification evaluation formula and error rate evaluation formula, and therefore does not increase the number of procedures and parameters, and (4) it is based on the percentages of case and control in data sets for each combination of genotypes and reveals more information regarding the effect of certain genotype combinations on a disease risk since the quantitative value of the ratios represents better classifications results.

The general MDR method does usually not consider imbalanced data set. However, many real data sets show ratios of cases and controls that are not 1∶1 but often higher than 1∶3. This large difference between cases and controls results in the model being biased and favoring low-risk groups. This larger number of low-risk groups invariably causes the model to shift attention to the more uninteresting aspects. Hence, the results of the general MDR would not be used to analyze the gene-gene interactions responsible for diseases and cancers. To overcome this problem, this study successfully used two functions to improve the MDR method and provide a powerful analysis tool that can investigate multiple-locus interactions in chronic dialysis patients. The improved performance of MDR-ER in generating significant models can potentially be applied to determine the complex gene-gene interactions among the huge number of SNPs involved in genome-wide association studies when data sets are imbalanced.

## Supporting Information

File S1 **Supporting file containing the following: Example.** The computational details of a different factor combination: SNPs(40, 56), and compare the difference between MDR-E and MDR-ER. **Figure S1.** Sample distributions in the SNPs(40, 56). **Figure S2.** The ratio between cases and controls in each cell. (A) Results using equation 1 (MDR-E); (B) Results using equation 2 (MDR-ER). **Figure S3.** Classification results. (A) Classified results of MDR-E; (B) Classified results of MDR-ER. **Figure S4.** Total number of high- and low-risk among the cases and controls. (A) results of MDR-E;(B) results of MDR-ER. **Table S1** Computation of all genotype combinations in SNPs(40, 56). **Table S2** Analysis results of the chronic dialysis data sets in 1∶1 cases and controls. **Table S3** Analysis results of the chronic dialysis data sets in 1∶2 cases and controls. **Table S4.** Analysis results of the chronic dialysis data sets in 1∶3 cases and controls.
(DOC)
