# Supplementary material for: MDR-ER: Balancing Functions for Adjusting the Ratio in Risk Classes and Classification Errors for Imbalanced Cases and Controls Using Multifactor-Dimensionality Reduction
Source: PLoS One. 2013 Nov 13;8(11):e79387. doi: 10.1371/journal.pone.0079387 (PMC3827354; doi:10.1371/journal.pone.0079387)
Supplement: File S1 — Supporting file containing the following: Example. The computational details of a different factor combination: SNPs(40, 56), and compare the difference between MDR-E and MDR-ER. Figure S1. Sample distributions in the SNPs(40, 56). Figure S2. The ratio between cases and controls in each cell. (A) Results using equation 1 (MDR-E); (B) Results using equation 2 (MDR-ER). Figure S3. Classification results. (A) Classified results of MDR-E; (B) Classified results of MDR-ER. Figure S4. Total number of high- and low-risk among the cases and controls. (A) results of MDR-E;(B) results of MDR-ER. Table S1 Computation of all genotype combinations in SNPs(40, 56). Table S2 Analysis results of the chronic dialysis data sets in 1∶1 cases and controls. Table S3 Analysis results of the chronic dialysis data sets in 1∶2 cases and controls. Table S4. Analysis results of the chronic dialysis data sets in 1∶3 cases and controls. (DOC) [file pone.0079387.s001.doc]

**Supplementary file**

# MDR-ER: Balancing functions for adjusting the ratio in risk classes and classification errors for imbalanced cases and controls using multifactor-dimensionality reduction

### Cheng-Hong Yang^1^, Yu-Da Lin^1^, Li-Yeh Chuang^2*^, Jin-Bor Chen^3^, and Hsueh-Wei Chang^4,5*^

^1^ Department of Electronic Engineering, National Kaohsiung University of Applied Sciences, Kaohsiung, Taiwan, ^2^ Department of Chemical Engineering & Institute of Biotechnology and Chemical Engineering, I-Shou University, Kaohsiung, Taiwan. ^3^Division of Nephrology, Department of Internal Medicine, Mitochondrial Research Unit, Kaohsiung Chang Gung Memorial Hospital, Chang Gung University College of Medicine, Kaohsiung, Taiwan. ^4^Department of Biomedical Science and Environmental Biology, Kaohsiung Medical University, Taiwan. ^5^Cancer Center, Kaohsiung Medical University Hospital, Kaohsiung Medical University, Kaohsiung, Taiwan.

In the supplementary file, we show the computational details of a different factor combination: SNPs(40, 56), and compare the difference between MDR-E and MDR-ER. Figure S1 shows the sample distributions in SNPs(40, 56). The symbols AA and aa represent the two genotypes of SNP 40, and the symbols BB and bb represent the two genotypes of SNP 56. In the bar chart, the bar represent the total number in genotype combinations, and the left and right bar respectively denote the case and control groups. The four operations of MDR-E and MDR-ER are detailed in the diagram below.


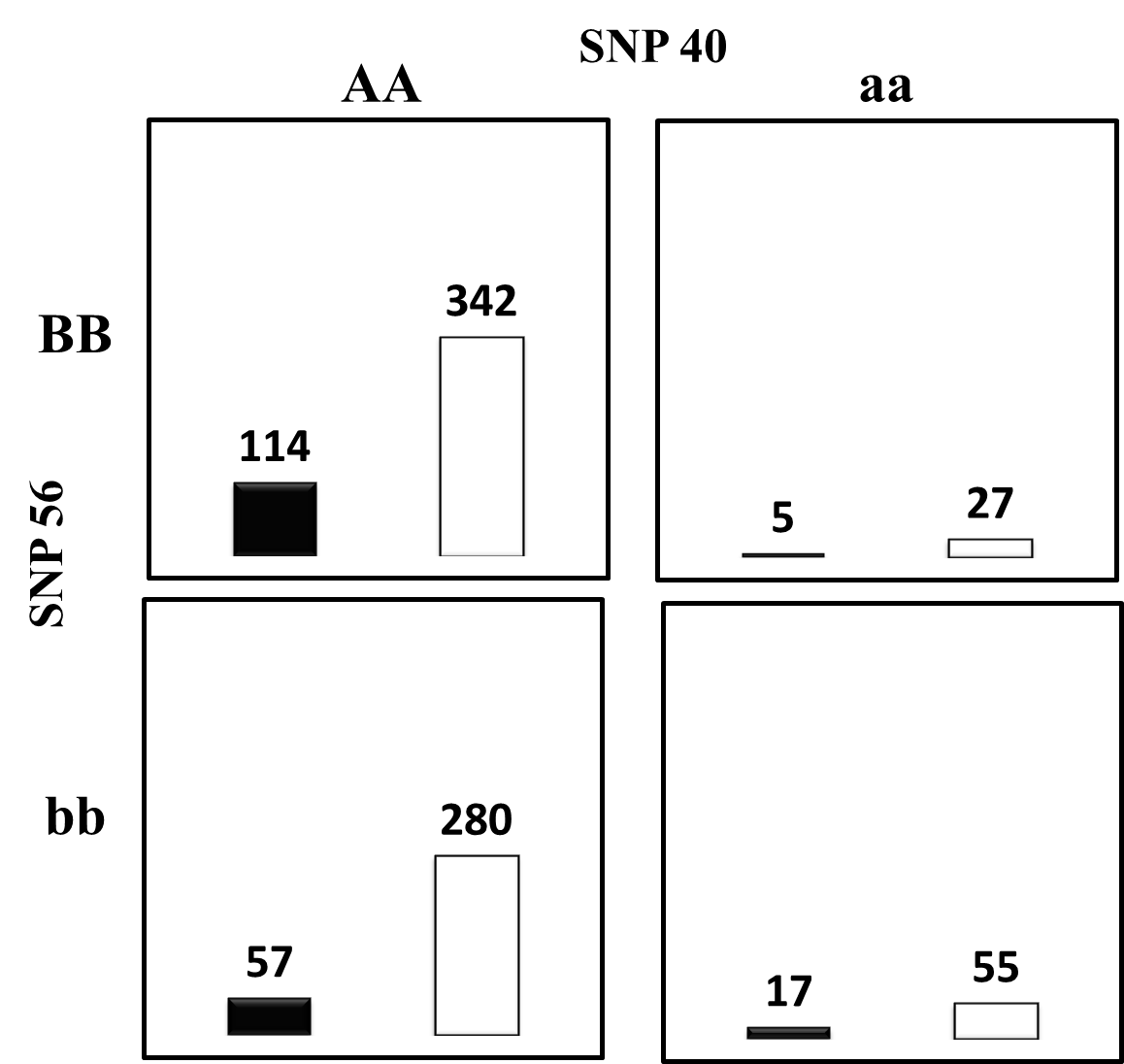


**Figure S1.** Sample distributions in the SNPs(40, 56).

1. Compute the ratio between cases and controls in each cell.

Equations 1 and 2 are the original ratio computation equation and our proposed ratio computation equation, respectively. The computational results are shown in Table S1. In Table S1, the genotype combination column lists all possible genotype combinations. The results shown in Figure S2.A and Figure S2.B are respectively computed by equations 1 and 2, i.e., MDR-E and MDR-ER.

**Table S1.** Computation of all genotype combinations in SNPs(40, 56).

| **Genotype combination** | **Equation 1** | | **Equation 2** |  |
| --- | --- | --- | --- | --- |
| AA-BB | 114/342 = 0.33 | | (114*704)/(342*193) = 1.22 |  |
| aa-BB | 5/27 = 0.19 | | (5*704)/(27*193) = 0.68 |  |
| AA-bb | 57/280=0.20 | | (57*704)/(280*193) = 0.74 |  |
| aa-bb | 17/55=0.31 | | (17*704)/(55*193) = 1.13 |  |
| $f(L)=\frac{\sum_{j=1}^{P^{*}} u(L,P_{j})}{\sum_{j=1}^{N^{*}} u(L,N_{j})}$ | | (1) | | |
| where $u(L,A)=\left\{\begin{matrix} 1 & \forall l\in A \\ 0 & \forall l\notin A \end{matrix}\right.\text{ },\forall l\in L$ | |  | | |
| $f(L)=\frac{N^{*}[\sum_{j=1}^{P^{*}} u(L,P_{j})]}{P^{*}[\sum_{j=1}^{N^{*}} u(L,N_{j})]}$ | | (2) | | |
| where $u(L,A)=\left\{\begin{matrix} 1 & \forall l\in A \\ 0 & \forall l\notin A \end{matrix}\right.\text{ },\forall l\in L$ | |  | | |


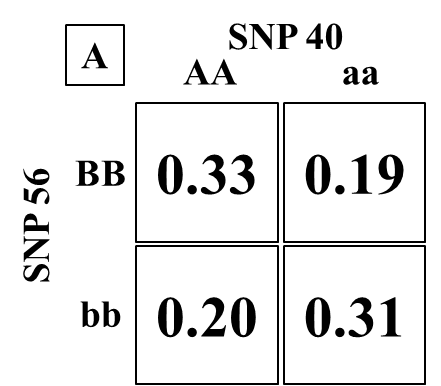

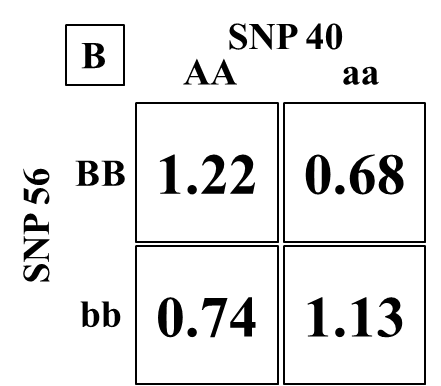


**Figure S2.** The ratio between cases and controls in each cell. (A) Results using equation 1 (MDR-E); (B) Results using equation 2 (MDR-ER).

2. Classification into high- and low-risk groups based on the ratio value

When the ratio value is bigger than a threshold of *T* = 1, the cell is classified as high-risk; otherwise, the cell is classified as low-risk. The classification results are shown in Figure S3. Figure S3.A shows the classified results of MDR-E, and Figure S3.B show the classified results of MDR-ER.


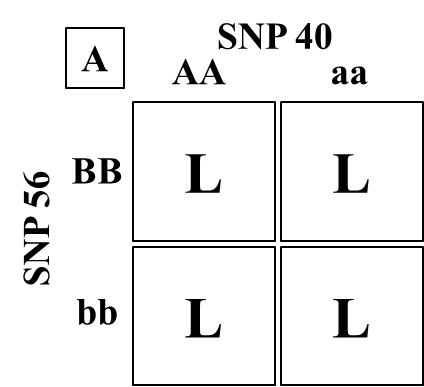

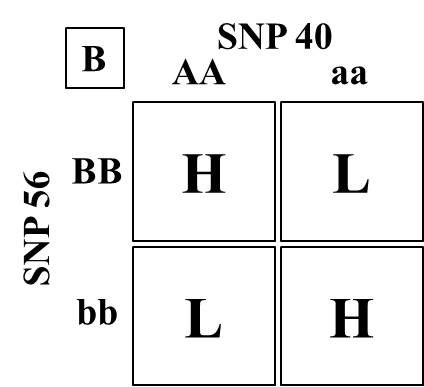


**Figure S3.** Classification results. (A) Classified results of MDR-E; (B) Classified results of MDR-ER.

3. Sum of the total number of high- and low-risk among the cases and controls.

Figure S4 show the sums of the total number of high- and low-risk among the cases and controls. The results of Figure S4.A are summed up based on Figures S1 and S3.A. The results of Figure S4.B are summed up based on Figures S1 and S3.B. For example, in Figure S4.B the high-risk group in the cases is summed up by the case numbers of AA-BB and aa-bb (114+17=131).


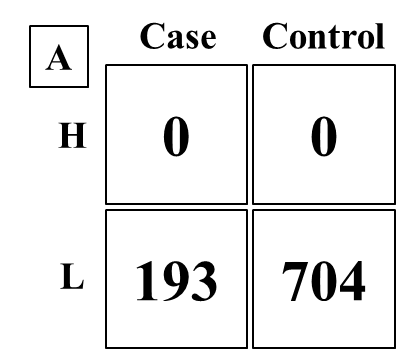

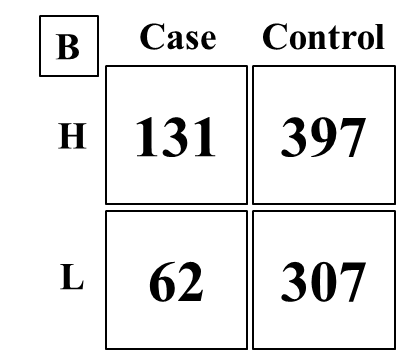


**Figure S4.** Total number of high- and low-risk among the cases and controls. (A) results of MDR-E;(B) results of MDR-ER.

4. Computation of error rate.

The error rates of MDR-E and MDR-ER are computed by equation 3. The error rates of MDR-E and MDR-ER are 0.5 and 0.443, respectively.

| $f(C)=0.5\times\left(\frac{FN}{TP+FN}+\frac{FP}{FP+TN}\right)$ | (3) |
| --- | --- |
|  |  |
| MDR-E： $\begin{matrix} f(C)=0.5\times\left(\frac{FN}{TP+FN}+\frac{FP}{FP+TN}\right) \\ \text{ }=0.5\times\left(\frac{193}{0+193}+\frac{0}{0+704}\right) \\ \text{ }=0.5 \end{matrix}$ | MDR-ER： $\begin{matrix} f(C)=0.5\times\left(\frac{FN}{TP+FN}+\frac{FP}{FP+TN}\right) \\ \text{ }=0.5\times\left(\frac{62}{131+62}+\frac{397}{397+307}\right) \\ \text{ }=0.443 \end{matrix}$ |

**Table S2.** Analysis results of the chronic dialysis data sets in 1:1 cases and controls

| Methods | Best candidate model | Consistency | TP | TN | Error rate | OR (95% CI) |
| --- | --- | --- | --- | --- | --- | --- |
| 2-locus |  |  |  |  |  |  |
| MDR-E | 56, 62 | 29/100 | 93 | 121 | 0.46 | 1.56 (1.04-2.35) |
| MDR-ER | 56, 62 | 29/100 | 93 | 121 | 0.46 | 1.56 (1.04-2.35) |
| 3-locus |  |  |  |  |  |  |
| MDR-E | 8, 52, 62 | 86/100 | 85 | 137 | 0.42 | 1.93 (1.26-2.93) |
| MDR-ER | 8, 52, 62 | 86/100 | 85 | 137 | 0.42 | 1.93 (1.26-2.93) |
| 4-locus |  |  |  |  |  |  |
| MDR-E | 17, 52, 59, 62 | 63/100 | 87 | 142 | 0.41 | 2.29 (1.49-3.50) |
| MDR-ER | 17, 52, 59, 62 | 62/100 | 87 | 142 | 0.41 | 2.29 (1.49-3.50) |
| 5-locus |  |  |  |  |  |  |
| MDR-E | 8, 31, 52, 59, 62 | 67/100 | 121 | 114 | 0.39 | 2.43 (1.61-3.65) |
| MDR-ER | 8, 31, 52, 59, 62 | 67/100 | 121 | 114 | 0.39 | 2.43 (1.61-3.65) |

**Table S3.** Analysis results of the chronic dialysis data sets in 1:2 cases and controls

| Methods | Best candidate model | Consistency | TP | TN | Error rate | OR (95% CI) |
| --- | --- | --- | --- | --- | --- | --- |
| 2-locus |  |  |  |  |  |  |
| MDR-E | 5, 64 | 92/100 | 22 | 374 | 0.46 | 4.01 (1.94-8.29) |
| MDR-ER | 45, 56 | 93/100 | 122 | 186 | 0.44 | 1.60 (1.12-2.78) |
| 3-locus |  |  |  |  |  |  |
| MDR-E | 5, 55, 64 | 96/100 | 27 | 372 | 0.45 | 4.32 (2.21-8.45) |
| MDR-ER | 45, 56, 77 | 88/100 | 121 | 199 | 0.43 | 1.79 (1.26-2.55) |
| 4-locus |  |  |  |  |  |  |
| MDR-E | 8, 21, 31, 65 | 33/100 | 23 | 365 | 0.47 | 2.35 (1.27-4.37) |
| MDR-ER | 8, 31, 59, 64 | 43/100 | 97 | 255 | 0.42 | 1.97 (1.38-2.80) |
| 5-locus |  |  |  |  |  |  |
| MDR-E | 8, 21, 31, 59, 62 | 79/100 | 54 | 339 | 0.42 | 2.80 (1.81-4.34) |
| MDR-ER | 8, 21, 31, 59, 64 | 68/100 | 80 | 300 | 0.40 | 2.47 (1.70-3.59) |

**Table S4.** Analysis results of the chronic dialysis data sets in 1:3 cases and controls

| Methods | Best candidate model | Consistency | TP | TN | Error rate | OR (95% CI) |
| --- | --- | --- | --- | --- | --- | --- |
| 2-locus |  |  |  |  |  |  |
| MDR-E | 55, 64 | 91/100 | 19 | 567 | 0.46 | 5.16 (2.46-10.84) |
| MDR-ER | 21, 64 | 58/100 | 105 | 333 | 0.44 | 1.62 (1.16-2.24) |
| 3-locus |  |  |  |  |  |  |
| MDR-E | 5, 55, 64 | 67/100 | 19 | 567 | 0.46 | 5.16 (2.46-10.84) |
| MDR-ER | 40, 56, 64 | 37/100 | 136 | 253 | 0.43 | 1.85 (1.30-2.63) |
| 4-locus |  |  |  |  |  |  |
| MDR-E | 5, 17, 43, 64 | 28/100 | 22 | 567 | 0.45 | 6.08 (2.95-12.54) |
| MDR-ER | 21, 59, 64, 71 | 90/100 | 108 | 353 | 0.42 | 1.98 (1.43-2.76) |
| 5-locus |  |  |  |  |  |  |
| MDR-E | 5, 17, 43, 55, 64 | 40/100 | 27 | 564 | 0.44 | 6.12 (3.18-11.77) |
| MDR-ER | 4, 21, 40, 56, 60 | 46/100 | 125 | 314 | 0.41 | 2.18 (1.55-3.05) |
